# Genetic and biochemical changes of the serotonergic system in migraine pathobiology

**DOI:** 10.1186/s10194-016-0711-0

**Published:** 2017-02-13

**Authors:** Claudia Francesca Gasparini, Robert Anthony Smith, Lyn Robyn Griffiths

**Affiliations:** 1 Menzies Health Institute Queensland, Griffith University Gold Coast, Parklands Drive, Southport, QLD 4222 Australia; 2 Genomics Research Centre, Institute of Health and Biomedical Innovation, School of Biomedical Sciences, Queensland University of Technology, Musk Ave, Kelvin Grove, QLD 4059 Australia

**Keywords:** Migraine with aura, Migraine without aura, Cortical spreading depression, Serotonin, Metabolomics, Melatonin, Tryptophan, Triptans, SERT, Kyneurine pathway

## Abstract

Migraine is a brain disorder characterized by a piercing headache which affects one side of the head, located mainly at the temples and in the area around the eye. Migraine imparts substantial suffering to the family in addition to the sufferer, particularly as it affects three times more women than men and is most prevalent between the ages of 25 and 45, the years of child rearing. Migraine typically occurs in individuals with a genetic predisposition and is aggravated by specific environmental triggers. Attempts to study the biochemistry of migraine began as early as the 1960s and were primarily directed at serotonin metabolism after an increase of 5-hydroxyindoleacetic acid (5-HIAA), the main metabolite of serotonin was observed in urine of migraineurs. Genetic and biochemical studies have primarily focused on the neurotransmitter serotonin, considering receptor binding, transport and synthesis of serotonin and have investigated serotonergic mediators including enzymes, receptors as well as intermediary metabolites. These studies have been mainly assayed in blood, CSF and urine as the most accessible fluids. More recently PET imaging technology integrated with a metabolomics and a systems biology platform are being applied to study serotonergic biology. The general trend observed is that migraine patients have alterations of neurotransmitter metabolism detected in biological fluids with different biochemistry from controls, however the interpretation of the biological significance of these peripheral changes is unresolved. In this review we present the biology of the serotonergic system and metabolic routes for serotonin and discuss results of biochemical studies with regard to alterations in serotonin in brain, cerebrospinal fluid, saliva, platelets, plasma and urine of migraine patients.

## Review

### Introduction

Migraine is a painful neurological disease whose manifestation results from an interplay of enviro-genomic factors and the severity of symptoms often propels sufferers to abuse medication. Migraine reigns as the 8^th^ most burdensome disease in the world and the 4^th^ most in women according to the 2012 Global Burden of Disease study [1]. The clinical features of migraine according to IHS classification include intense, pulsating head pain localized to one side of the head that can effectively disable its sufferer for up to 72 h [2]. Accompanying symptoms of migraine in addition to the headache include nausea, vomiting, and hypersensitivity to lights, sounds and/or smells, all of which can be aggravated by physical activity [2]. The disease is believed to develop in genetically vulnerable populations, disabling an estimated 12% of Caucasian people, varying in accordance with genetic background, climatic region, socioeconomic status, life-style, presence of other diseases and the general health of the sourced population [3, 4]. There is also a well-established overrepresentation of the disease in females, affecting ~3 times more females than males due to a complex interplay of hormonal and enviro-genomic exposures [5].

Migraine is diagnosed by specialized physicians utilizing the current International Headache Society (IHS) criteria (ICHD-3, beta version currently available at https://www.ichd-3.org/) [2]. Acceptance of these criteria has facilitated modern day molecular genetic research by standardising diagnosis of headache and headache-related disorders in absence of any clearly identifiable pathology, biomarkers and diagnostic tests. Migraine is classified into two main types, distinguished by the presence of a variety of sensory disturbances (termed aura) that can occur in the early stages of the headache: migraine with aura (MA) or migraine without aura (MO) [2]. Migraine is a complex disorder that evolves due to a combination of multiple genes and external factors such as gene-environment, gene-nutrient, gene-gene interactions and epigenetics that result in multiple presentations of the disorder making it difficult to pinpoint the relevant genomic risk factors [6].

Literature on the pathophysiology of migraine continues to evoke a disorder of neurovascular origin as demonstrated by the involvement of cranial neurovascular anatomical structures. This neurovascular scaffold encompasses three main domains: a neural domain (i.e., hyperexcitability and CSD-like neural events) [7, 8], vascular domain (intracranial constriction and vasodilation) and a nociceptive domain (activation of trigeminal structures, a network of neurons that sense pain signals and release of several ‘migraine mediators’, neuropeptides, such as CGRP). These three domains describe the main aspects of the current model of the pathobiology of migraine [9–11]. Genetic studies have focused on investigating genetic variability in multiple neurotransmitter systems which interact and overlap at different levels.

Neurotransmitters regulate intracellular signal transduction events as well as neural function, morphology and circuit formation [12]. Interruptions in intra- and interneuronal signalling due to inappropriate activation or inactivation of regulatory proteins, including nuclear transcription factors can alter the electrophysiological signalling of neurons resulting in structural and functional changes [13–15]. This hypothesis of ‘malfunctioning ion channels’ that set the electrical activity in nerve cells that form circuits modulating pain was revived by the discovery of mutations in the potassium channel TRESK [15]. Similarly, the recognition that the three main types of Familial Hemiplegic Migraine are caused by mutations in ion channels implies that ion channel dysfunction may be the primary cause for migraine symptoms in non-familial cases as well [16]. In this review we considered the biology of the serotonergic system and its metabolism and results of biochemical studies regarding the content of serotonin in the brain, cerebrospinal fluid (CSF), saliva, platelets, plasma and urine of migraine patients and we link the evidence together in a biochemical model that considers the application of metabolomics for guiding future research on the pathobiology of migraine.

### Serotonergic biology and genetics

Biochemical, genetic and pharmacological studies have investigated potential dysfunction of neurotransmitters in migraine susceptibility. The involvement of serotonin in migraine has been known for many years following Sicuteri’s observations of an increase of 5-hydroxyindoleacetic acid (5-HIAA), the main metabolite of serotonin, in the urine of 15 patients during migraine attacks [17]. This was the first indication of an anomaly of serotonin metabolism in migraineurs and since then, many scientific research studies have focused on the genetic components of the serotonergic system (see Table 1). Receptors, transporters and enzymes are the key molecular mediators that regulate and maintain serotonin levels in the brain and periphery. Serotonin is distributed in neuronal and several non-neuronal tissues of the cardiovascular system, gastrointestinal, renal systems and the blood [18]. Serotonin regulates a wide array of physiological functions via a specific set of receptors which are mostly G protein-coupled receptors (except receptor 5HT3 which is a ligand gated cation channel) that activate an intracellular second messenger cascade to mediate excitatory or inhibitory neurotransmission [19]. Serotonin receptors have been localized in vestibular and trigeminal ganglion cells in monkey [20].

**Table 1:** Genetic components of the serotonergic system

Enzyme	Gene	Locus	Function	Significance for migraine
Tryptophan hydroxylase 1	TPH1	11p15.3-p14	TPH1, peripheral form, converts tryptophan to 5 hydroxy tryptophan, rate-limiting step in the synthesis of 5-HT, determines the availability of 5-HT and its rate of production.	TPH enzymes regulate brain-specific serotonin deficiency, weak association [43].
Tryptophan hydroxylase 2	TPH2	12q21.1	TPH2, neuronal form, synthesis of serotonin.	
Monoamine oxidase A	MAO-A	Xp11.3	MAO-A, outer mitochondrial membrane, oxidative deamination of amines, such as dopamine, norepinephrine, and serotonin.	Enzymes metabolise triptans.
Monoamine oxidase B	MAO-B	Xp11.23	MAO-B, both subtypes have a widespread occurrence in the brain and in peripheral tissues.	MAO inhibitors promote the accumulation of monoamines and reduce uncontrolled vasodilation, and were used in treating migraine but discontinued due to side effects [73].
Amino acid decarboxylase	DDC	7p12.2	DDC, catalyses the decarboxylation of L-5-hydroxytryptophan to 5-HT and L-tryptophan to tryptamine.	Gene variations have been investigated in relation to neuropsychiatric disorders.
Aldehyde dehydrogenase 2	ALDH2	12q24.2	ALDH2, located in mitochondria produce 5-hydroxyindole acetic acid as the major excreted metabolite of serotonin.	
Receptor Protein	Gene	Locus	Potential and mechanism	Significance for migraine
5HT1	HTR1A	5q11.2-q13	Inhibitory, decreasing cellular levels of cAMP	
	HTR1B	6q13		Co-localization of 5HT1_B_, 5HT1_D_ and HTR1_F_ receptor subtypes in vestibular ganglia [20, 74]. Triptans have high affinity for serotonin receptors.
	HTR1D	1p36.3-p34.3		
	HTR1E	6q14-q15		
	HTR1F	3p12		Lasmiditan is a 5-HT1F receptor agonist by non-vascular mechanism [75].
5HT2	HTR2A	13q14-q21	Excitatory, increasing cellular levels of IP3 and DAG	Cortical density of the excitatory 5-HT2 receptor, which is involved in pain processing, is not altered interictally in migraine patients [76].
	HTR2B	2q36.3-q37.1		
	HTR2C	Xq24		Positive association in SNP in HTR2C promoter in Turkish population with migraine [40].
5HT3	HTR3A	11q23.1	Excitatory, depolarizing plasma membrane	Role in facilitation of inflammatory and neuropathic pain [77].
	HTR3B	11q23.1		
	HTR3C	3q27.1		
	HTR3D	3q27.1		
	HTR3E	3q27.1		
5HT4	HTR4A	5q31-q33	Excitatory, increasing cellular levels of cAMP	Central 5-HT4 receptor binding might serve as a biomarker of serotonergic tonus in the human brain [78].
5HT5	HTR5A	7q36.1	Inhibitory, Decreasing cellular levels of cAMP	
	HTR5B	2q14.1		
5HT6	HTR6A	1p36-p35	Excitatory, increasing cellular levels of cAMP	
5HT7	HTR7A	10q21-q24	Excitatory, increasing cellular levels of cAMP	5-HT7 receptor activation promotes NMDA-evoked currents [79, 80]. 5-HT7 receptor has differing roles in peripheral versus central pain mediation and is clinically relevant for the treatment of depression, a migraine co-morbidity [81].
Transporter protein	Gene	Locus	Function	Significance for migraine
Serotonin transporter, SERT	SLC6A4	17q11.2	Reuptakes serotonin from the synapse, role in psychiatric disease	Meta-analyses by the groups of Liu and Schurks report a positive association with SERT and migraine [27, 28].
Plasma membrane monoamine transporter, PMAT	SLC629A4	7p22.1	Transport of both serotonin and dopamine	

*5-HT* 5-hydroxytryptamine, *cAMP* cyclic adenosine monophosphate, *IP3* inositol trisphosphate, *DAG* diacylglycerol, *SNP* single nucleotide polymorphism

Note: PMAT (Plasma membrane monoamine transporter) is involved in the transport of both serotonin and dopamine

Current research on the biology of the serotonergic system in disease is focusing on genetic alterations in synaptic and post-receptor signalling proteins that affect serotonergic neurotransmission by increasing or decreasing serotonin’s actual or effective availability in the CNS (See Table 1) [21]. Genetic variability at the level of gene transcription, mRNA processing and translation or post-translational modification as well as intracellular trafficking in the genetic components of the serotonergic system may generate gene products that may lead to structural and functional changes in brain circuits and provoke disease [22]. The two most studied components of the serotonergic system that have come into the spotlight in determining susceptibility to migraine as well as multiple neuropsychiatric disorders are the serotonin transporter (SERT, also known as SLC6A4), which reuptakes serotonin from the synapse, and monoamine oxidase A (MAOA), an important enzyme that degrades serotonin and metabolizes triptans [23]. Both these proteins are important in regulating levels of serotonin in the brain and carry polymorphisms in their promoter regions that cause differential transcriptional activity and thus impact drug metabolism [22, 24–26].

Several association studies have investigated genetic variants that may alter the functions of genes involved in serotonin functionality and regulation. The serotonin transporter gene *SERT* has been extensively studied. In this gene, two polymorphisms have been of particular interest. The first polymorphism consists of a 17 bp variable number of tandem repeats known as (STin2 VNTR) in intron 2 with two common alleles (STin2.10 and STin2.12) composed of 10 or 12 repeat units respectively [27]. To get a clearer picture, Schurks et al., [27] considered five studies that evaluated the 5-HTT VNTR STin2 polymorphism and concluded the STin2 12/12 genotype may be influential in determining migraine susceptibility among populations of European descent. A further meta-analysis by Liu et al., [28] reviewed 15 studies and found that the 5-HTT VNTR STin2 12/12 genotype confers an increased risk for migraine in the general population.

The second polymorphism is a 44-bp insertion/deletion functional polymorphism in the promoter region known as 5-HTTLPR. There are two common allelic forms, and the 14 repeat containing short variant (S) is associated with slower clearing of serotonin from the synaptic cleft [29]. Analysis of this polymorphism has provided conflicting results. The S allele has shown association with migraine in some studies [30–33] while in other studies no evidence of association was identified [34, 35]. In a meta-analysis of 10 studies of Europeans and Asians Schurks et al., reported no overall association between the 5-HTTLPR polymorphism and migraine, although gender and migraine aura status may influence results among Europeans [29]. Although various polymorphisms in serotonin related genes have showed altered allelic distribution in different migraine populations there has been, to date, insufficient evidence to confirm a specific serotonin receptor gene being directly associated with the disorder [36, 37].

Genetic studies continue to provide new data regarding the involvement of serotonin in migraine and in particular support the idea that migraine is a polygenic disorder as recently reported in a large meta-analysis of 375,000 individuals by Gormley et al., [38] that identified 38 susceptibility loci for migraine. Thompson et al. identified a positive association at the *5-HT1D* receptor locus in 64 extended families with migraine with aura [39]. The locus 1p36 for the *5-HT1D* gene is a candidate locus because it is a target of triptan drugs and it was also considered in the context of the association of PRDM16 in MO which is located proximally to 1p36. Recently, a significant association was identified between migraine and a functional polymorphism rs3813929 localised in the promoter region of the serotonin receptor gene 5-HT2C [40]. The T allele of this polymorphism affects the transcription rate of the 5-HT2C receptor and was more common in a Turkish population with migraine [41].

Additionally, genetic research has explored serotonin synthesis and its role in migraine. Serotonin is synthesized in a short metabolic pathway consisting of two enzymes: tryptophan hydroxylase (TPH) and amino acid decarboxylase (DDC) (See Table 1) [42]. The TPH2 gene is an obvious candidate to genotype because in theory low activity of this enzyme may decrease the amount of serotonin available in the brain. However, a study by Jung et al., [43], did not find a positive association with migraine for two SNPs (rs1487275 and rs1386486) in the TPH2 gene (TPH1 is the peripheral form, TPH2 is the neuronal form) in a population of 503 migraineurs and 515 healthy controls [43]. Although this study does not support TPH2 as a key player in the migraine circuit TPH2 is emerging as a therapeutic target for stress disorders and abnormalities in TPH activity have been implicated in a variety of psychiatric disorders [44–46]. Conceivably, there may be other effects, including epigenetic, gene regulatory and or expression effects affecting this locus yet to be determined [44]. These data together with TPH2 knockout mice have provided additional insights into the role of TPH enzymes in regulating brain-specific serotonin deficiency [47–49].

#### Migraine therapy and triptans

Triptans are currently the most effective drugs in treating migraine and are the supporting pillar of migraine therapy [50]. Triptans amplify the serotonin signal by stimulating serotonin receptors located in cranial blood vessels and nerve endings and relieve pain by constricting blood vessels and inhibiting the release of peptides, including CGRP [51] and substance P as well as acting in other ways not yet known [52]. At the cellular level triptans alleviate migraine symptoms by binding to serotonin 5-HT1b, d and f receptors along pain signaling circuits and are effective due to high receptor specificity and disease specificity [53]. Recent studies [54–56] continue to reveal triptans complex actions including meta-analyses comparing the effectiveness of triptans alone or in combination with other drugs [57]. Although triptans are the most effective drugs for migraine they exhibit therapeutic variability in different patient groups. The different levels of drug response can be explained by different metabolic routes, rates and efficiency.

In a meta-analysis by Cameron et al., 133 randomized controlled trials were included and the conclusion from this study was that triptans are generally better than ergots and provide equal or better pain relief when compared with non-steroidal anti-inflammatory drugs (NSAIDs), acetylsalicylic acid (ASA) and acetaminophen [57]. Triptans used in combination with ASA or acetaminophen, or by alternative administration methods (injectables) yielded slightly better outcomes than standard dose triptan tablets. Thorlund et al., evaluated the efficacy of seven triptans in a multiple treatment comparison meta-analysis by examining data from 74 double-blinded randomized clinical trials comparing triptans to either placebo or to another triptan [58]. Among the seven different triptans tested, eletriptan was the best triptan and all triptans were found better than placebo [58]. This result was also upheld by a recent report showing that triptans still have the most favorable efficacy-tolerability profile [59].

The hunt for better more specific drugs continues particularly in the era of pharmacogenetic research whose goal is to evaluate the impact of genetic constitution on migraine drug response [60]. Pharmacogenetics recognizes that numerous genes (drug metabolizing enzymes and drug transporters) play a role in the mechanism of drug response, resistance, toxicity, drug transport or drug targeting and ultimately affect how patients metabolize and consequently respond to different drug regimens [61, 62]. This information can help inform doctors prescribing practices ie. responders versus non-responders, avoiding adverse drug reactions, optimizing drug dose for the individual and ultimately guide prescription of the ‘right’ triptan to the ‘right’ patient [63].

In a study by Terazzino et al., the STin2 VNTR polymorphism in the SERT gene was shown to be associated with a higher risk of inconsistent response to triptans in migraine patients [64]. However the authors caution some limitations in their study such as a small sample size but nevertheless support future pharmacogenetic testing to identify algorithms for more effective treatment [64]. Recently, Christensen et al. identified a single risk variant, rs2651899 in the PRDM16 gene among 12 single nucleotide polymorphisms (SNPs) genotyped chosen based on prior GWAS findings by Antilla [65] to be significantly associated with efficacy of triptans [60]. To explore the role of genetic factors in drug metabolism Gentile et al., 2010 genotyped polymorphisms in CYP450 and MAOA, genes that are involved in Phase I metabolic reactions, and in target mechanisms (GNB3) of triptans and reported a significant correlation between MAOA uVNTR polymorphism and a chronic migrainous population [66, 67]. Serotonin receptors are also targeted by other drugs besides triptans and in a study by Brandl et al., polymorphisms in serotonergic genes (HTR2A, SERT) were found to be associated with antipsychotic treatment response [68].

In addition to pharmacogenomics a new paradigm has emerged ‘pharmacometabolomics’ for characterizing the metabolic (pharmacokinetic and pharmacodynamic) response to a drug taking into account both environmental and genomic factors [69]. Although there is still a paucity of studies in this domain, recent studies are using both metabolomic and genomic data to study selective serotonin reuptake inhibitor (SSRI) treatment outcomes in patients with major depressive disorder (MDD) by use of a “pharmacometabolomic-informed” pharmacogenomic research strategy [70]. In a follow up study in 2016, Gupta et al., uncovered two main SNP signals in two candidate genes (TSPAN5 and ERICH3) which were associated with the clinical response to SSRI therapy in MDD patients [71]. Functional genomic studies assessed serotonin pathway enzyme expression after knockdown (KD) or over-expression (OE) of TSPAN5 and ERICH3 in neurally-derived cell lines by qRT-PCR and quantitative Western blot. KD of TSPAN5 was associated with a significant decrease of mRNA and protein levels for the serotonin pathway enzymes TPH1, TPH2, DDC, MAOA, as well as the serotonin transporter SLC6A4. Both TSPAN5 and ERICH3 proteins affect serotonin concentrations in cell culture media. The identification of ERICH3 and TSPAN5 in the regulation of serotonin or variation in SSRI response is novel and recent studies have reported TSPAN5 may also promote Notch signalling [72].

Although specific SNPs in genes that metabolize triptans have yet to be identified, current pharmacogenetics studies indicate that defining the effect SNPs have on drug metabolism and treatment response may have value in classifying patients into groups of poor metabolizers to predict therapeutic responses and to evaluate sex differences. Several studies have shown that SNPs in genes of most metabolic enzymes can affect enzyme activities and may account for inter-individual variation, in drug bioavailability and efficacy and consequently can bear a role in therapy response [63]. Results of future testing the clinical utility of SNPs and genetic variability in the signalling components of the serotonergic system as well as additional genes involved in triptan metabolism will impact patient care and may be useful to personalize medicine.

#### Metabolic routes of serotonin

##### Tryptophan - Indole-kynurenine-niacin pathway

Tryptophan is a requisite precursor necessary for various metabolic reactions primarily the synthesis of serotonin, proteins, melatonin, tryptamine and kynuramines [82, 83]. In the body, tryptophan is metabolized, via two basic pathways, the indole-kynurenine-niacin pathway and the serotonin-melatonin pathway [84]. The majority of tryptophan, 99% is metabolized and degraded along the kynurenine pathway which generates neuroactive compounds collectively called the kynurenines that antagonize the NMDA receptors [85]. Several studies of neurological diseases have reported kynurenine pathway (KP) metabolites and/or enzyme activity to be up- and/or down-regulated in relation to disease [86, 87]. Recently Curto et al., identified an association of fluctuations of kynurenine metabolites in serum samples from 21 cluster headache patients [88]. Curto et al., also identified altered kynurenine pathway metabolites in serum of chronic migraine patients [89]. Indeed activation or inhibition of kynurenine pathway enzymes and accumulation of kynurenines within the CNS has been considered a therapeutic strategy [90]. However, the potential of this therapeutic strategy has not yet been tried in migraine [91].

To investigate central and peripheral serotonin turnover in neurological disorders and in depressed mood, researchers have adopted the tryptophan depletion technique (Acute Tryptophan Depletion ATD) [82]. Initial studies in healthy volunteers showed that depletion of tryptophan is correlated with a decline in central serotonin turnover [92, 93]. The tryptophan-serotonin relationship has been confirmed in animal models [94–96] and was first demonstrated in 1970, indicating this mechanism may be potentially important in serotonin related pathologies in humans [97, 98]. Changes in the composition of the blood plasma in the periphery can influence functioning of the nervous system and affect various serotonin-dependent brain functions and mood states like depression [99–101]. Changes in diet and nutrition can lead to changes in brain levels of the precursors for neurotransmitter synthesis and influence the rates at which neurons synthesize neurotransmitters [101]. Tryptophan depletion increases nausea, headache and photophobia in migraine sufferers [102]. Two studies in the 70s, tested L-tryptophan treatment in headache patients and reported headache indices were markedly lower with supplementation of L-tryptophan than with placebo [103, 104]. The utility of tryptophan for both research and clinical purposes has been underexplored. Further analysis of the relationship of plasma tryptophan with brain serotonin metabolism in both normal and diseased states will be beneficial to understanding the complex serotonergic biochemistry of migraine.

##### Tryptophan - Serotonin-melatonin pathway

Melatonin is a hormone that is synthesised at night-time from the precursor tryptophan in the pineal gland. Endogenous melatonin plays an important function in regulating circadian rhythms for proper sleep-wake cycles as well as many neuronal and hormonal functions [105, 106]. Multiple studies have demonstrated altered levels of melatonin secretion at night in the plasma or urine of migraine patients with cluster headache, migraine with and without aura, menstrual migraine, and chronic migraine relative to controls that spurred further investigation of melatonin as a prophylactic therapy [107–113]. Melatonin has also been found to be altered in other central nervous system (CNS) disorders, such as stroke, obsessive-compulsive disorder, mood and schizophrenia [114, 115]. Melatonin has regulatory effects on gastrointestinal tract motility and sensation, has sleep promoting effects and mood regulation and anti-stress effects [116]. Consequently, it is not surprising that melatonin supplements have shown potential in diminishing pain intensity in migraine co-morbid conditions such as fibromyalgia and irritable bowel syndrome [116, 117].

Melatonin has been considered to hold therapeutic properties on headache profiles due to its normalizing effects on circadian rhythms and sleep and was further pursued in controlled trials because it is non-toxic, low cost and easily available over the counter in most countries (see Table 2) [118, 119]. Bougea et al., [120] conducted the longest trial lasting for a period of 6 months and demonstrated a positive effect in a children patient group. Studies on melatonin use in migraine have had some limitations, such as lack of adequate control and placebo, had a small sample size and throughout some of the trials the patients were permitted to take rescue medication for symptomatic relief of migraine during the trial period, limiting the comparisons that can be made. The future of melatonin-based therapy may also include melatonin receptor agonists and the design of trials with a longer follow up period, standardized designs, controls and IHS guidelines to establish the utility of melatonin-based therapy as a prophylactic therapy [121].

**Table 2:** Summary of trials of melatonin-based therapy for migraine prophylaxis

Reference	Study design	Cases and migraine type	Daily dose and duration	Primary endpoint	Result
Peres et al., 2004 [122]	open-labelled	Total adults, 32	3 mg/day, 30 min before bedtime for 3 months	Percentage of patients with >50% reduction in migraine frequency	positive
Miano et al., 2008 [123]	open-labelled	Total children, 22 13 MO 1 MA 8 CTTH	3 mg/day for 3 months	Reduced the frequency of headache attacks by >50% from baseline	positive
Alstadhaug et al., 2010 [124]	randomized, double-blind, placebo-controlled crossover study	Total adults, 46 25 MO 13 MA	2 mg/day prolonged release melatonin, given 1 h before bedtime for 2 months	Migraine attack frequency (AF)	negative
Fallah et al., 2015 [125]	open-labelled, non-randomized, uncontrolled	Total children, 60 32 MO 28 MA	0.3 mg/kg for 3 months	Percentage of patients with >50% reduction in migraine frequency	positive
Bougea et al., 2016 [120]	open-labelled	Total children, 41 12 TTH 37 M	4 mg/day 30 min before bedtime for 6 months	Reduced headache frequency	positive

*CCH* chronic cluster headache, *ECH* episodic chronic headache, *TTH* tension-type headache, *CTTH* chronic tension-type headache, *MA* migraine with aura, *MO* migraine without aura, *M* migraine

##### Tryptophan and tryptamine

Tryptamine is another functional neuromodulator produced from tryptophan metabolism and structurally related to serotonin [126, 127]. D’Andrea et al., [115] identified low levels of tryptamine in the plasma of 73 chronic migraine CM patients and 13 chronic tension-type headache (CTTH) patients relative to a group of 37 healthy subjects [128]. In contrast the levels of circulating 5-HT and 5-HIAA were within normal range. The low tryptamine plasma levels found in CM and CTTH patients lead the authors to the conclusion that these two primary chronic headaches are characterized by “neurotransmitter and neuromodulator metabolic abnormalities in a hyperexcitable and hypoenergetic brain” due to insufficient serotonergic control of the pain threshold [129]. The role of tryptophan and tyrosine metabolism in migraine headache is reviewed in D’Andrea et al., [129–131].

#### PET imaging studies of serotonin

In the study of neurological disorders such as migraine, a recognizable limitation with investigating *in vivo* the biochemistry of CNS functioning is access to neuronal brain tissue of affected patients for controlled experimental study [132]. In view of the non-existence of high-quality post-mortem brain tissue samples to study CNS functioning *in vivo,* peripheral blood is being proposed as a valid surrogate for genetic studies of migraine [133], along with cerebrospinal fluid, saliva, platelets, plasma and urine to study neurotransmitter function. Given that migraine is a complex multi-faceted disorder, it makes sense to take a multi-dimensional approach that considers the whole organism.

The continued development and increasing availability of positron emission tomography (PET) and single positron emission computerized tomography (SPECT), and radio-ligands has enabled the study of serotonergic biology *in vivo* [134, 135]. Due to high sensitivity and better anatomic resolution, imaging techniques have provided an entry point to study perturbed functional networks in neurologic and psychiatric disorders [136] and obtain more information about the distribution, density and activity of single receptor molecules in relation to migraine-specific actions of triptans [137]. Three studies, have thus far employed the non-invasive method [138] PET imaging using the tracer α-[^11^C] methyl-L tryptophan (^11^C-AMT) for measuring serotonin synthesis in the human brain [139]. This method is based on the regional trapping of tracer ^11^C-AMT concentration and uses *in vivo* measurements of ^11^C-AMT plasma with reference to the brain trapping constant (K*, mL/g/minute) [138]. Using this method, Chugani et al., [127] reported a trend of elevated serotonin synthesis capacity in three female patients with MO after migraine patients were treated prophylactically with beta-adrenergic antagonists propranolol or nadolol, thought to be due to antagonism of the 5-HT1A receptor on serotonergic neurons [140]. Additionally, two other PET studies demonstrated that serotonin receptors/transporters can be upregulated interictally (in between attacks) in migraine [141, 142].

Sakai et al., performed a similar study in six migraine patients with PET imaging, scanning participants during an attack, after administering sumatriptan and in between attacks [143]. Statistically significant changes were reported only in the migraine group treated with sumatriptan. Serotonin synthesis was significantly reduced, with a 33% reduced uptake of ^11^C-AMT in a majority of brain regions, similar effects were also reported in rats [143, 144]. The observed decrease in serotonin synthesis could be the body’s physiological adaptation to the presence of triptan which mimics the effect of serotonin. In a recent study, Sakai et al. tested the hypothesis of serotonergic dysfunction in migraine by scanning six female migraine subjects with MO at baseline and after oral administration of 40 mg of eletriptan, the triptan with the highest lipophilicity [145]. In the migraine group who received eletriptan, a significantly reduced (16.3%) global K* value for serotonin was obtained in the migraine subjects in the interictal phase of their condition [145]. Park et al., in a pilot study of eight female migraine patients investigated the availability of SERT in the brain stem and concluded that migraineurs who experience more painful headaches have lower serotonin neurotransmission and additionally reported an age-related decline of SERT availability [146]. In summary, the results from these studies suggest that brain serotonin synthesis rate may be altered in migraineurs and that triptans are effective on pain pathways by decreasing brain serotonin synthesis.

#### Biochemical studies of serotonin in CSF and saliva

The underlying hypothesis motivating studies of CSF is that because CSF is in direct contact with the brain interstitial fluid, serotonin and biochemical changes in the brains of migraine patients may best be reflected in this fluid [147]. Studies examining CSF are few, however, because they require spinal puncture, a costly and invasive procedure. Two studies, measuring serotonin activity in CSF can be found, one by Barrie et al., [135] which indicated no change in 5HIAA levels in CSF of migraine patients with respect to the controls and [148] one study by Kangasni et al., [149] reported an increase in 5HIAA levels in CSF (see Table 4).

Saliva has been used as a diagnostic tool to evaluate neurotransmitter function because it contains a variety of neuropeptides, integrating with the trigeminovascular and neuroendocrine systems [150–152]. There have been more studies trying to use saliva in the diagnostic and therapeutic assessment of migraine, e.g. the studies of Cady et al. [153] and Bellamy et al. [154] on CGRP in saliva of migraineurs. To date only one study by Marukawa et al., reported higher concentrations of serotonin and substance P in TTH patients [155]. The clinical usefulness of saliva has not yet been established, additional studies may be able to develop protocols able to make use of this easily accessible fluid, perhaps through correct timing of sampling [156].

#### Biochemical studies of serotonin in platelets

Interest in investigating platelets and peripheral biochemical factors is based on their interaction and proximity with the vascular endothelium. The vascular endothelium is a metabolically and physiologically active tissue that is semi-permeable and is important in regulating vascular tone [157]. Neurotransmitters and other vasoactive agents capable of modulating the nerve and vascular systems are actively produced by the vascular endothelium and can disrupt endothelial function [158]. Complex functional interactions that cause changes in endothelial function, vascular oxidative stress, promote platelet activation and enhance the inflammatory process can change the coagulant properties of blood producing alterations in neurovascular function and platelets may be an important part of these processes [159–162]. Attention has turned to the role of platelets in migraine due to the association and co-morbidity of migraine with several cerebrovascular disorders, including arterial dissection, ischemic stroke, and cardiovascular disease as well as the increased risk of thromboembolic events and the favourable effects of antiplatelet medication [163, 164].

Platelets carry the largest reserve of serotonin which they capture from enterochromaffin cells [165]. In the gastrointestinal tract, serotonin acts as a key signalling molecule mediating many GI functions, including peristalsis, secretion, vasodilation and perception of pain or nausea, vomiting, which are common symptoms experienced by migraineurs [166–168]. Additionally, platelets express SERT proteins (membrane-bound serotonin-selective reuptake transporter) that are identical to brain SERT [169]. Due to its role in clearing serotonin from the synaptic cleft, SERT plays a central role in neurotransmission and a broad role in brain activity making it a sought after therapeutic target of multiple neuropsychiatric disorders [170]. SERT knockout mice display notable reduction (60–80%) in serotonin concentration in several brain regions [171] and markedly depleted serotonin tissue stores [172–174].

Numerous studies have found an association between migraine and a dysfunction in platelets (see Tables 3 and 4). The platelet changes that have been described in migraineurs include increased platelet-to-platelet aggregation and up-regulation of platelet-leukocyte aggregates. Morphological differences in platelets of migraineurs reported include: 1) containing more ADP; 2) having more dense granules and; 3) showing qualitative differences in their serotonin release reaction and clumping [175]. The contemporary literature is not conclusive on the relationship between platelet serotonin content and migraine however it generally agrees that the distorted behaviour of platelets presents with subtle changes in various functions (reviewed in [176]). In a case study of two sisters with hemiplegic migraine, low levels of systemic serotonin, including the CSF serotonin metabolite 5-hydroxyindoleacetic acid and low platelet serotonin levels were described [177]. Interestingly, administration of 5-hydroxytryptophan (a precursor of serotonin) and carbidopa ameliorated the sisters’ clinical symptoms.

**Table 3:** Small scale biochemical studies of platelet serotonin in migraine patients and controls

Reference	Substance	Material and methods	Cases/ Controls	Results
(Dalsgaard-Nielsen et al., 1974) [210]	5-HT	5-HT in platelets estimated by Udenfriends method	M = 8 NC = 10	No change in the uptake or the endogenous release of 5-HT
(Kalendovsky , 1975) [211]	5-HT	Platelets from whole venous blood	M = 19 NC = 23	Migraine patients demonstrate platelet hyperaggregability more than normal subjects to all aggregating agents tested
(Dvilansky et al., 1976) [184]	5-HT	Platelets	MO = 22 MA = 16	Plasma during attacks released significantly more 5-HT
(Muck-Seler et al., 1979) [212]	5-HT	Platelet poor-plasma (PPP) using spectrophotofluorimetric method of Crawford and Rudd	M = 24 NC = 25	Unchanged 5-HT platelet level during migraine attack
(Rolf et al., 1981) [213]	5-HT	Platelets	MCH = 23 NC = 20	↓ 5-HT observed in patients with muscle contraction headache
(Carroll et al., 1982) [214]	5-HT	Platelets	MO = 25 NC = 18	↓ reduced transport of 5-HT in platelets
(Oxman et al., 1982) [215]	5-HT	Platelets	M = 16 NC = 10	Membrane lipid composition may play a role in disease
(Launay et al., 1982) [216]	5-HT	Platelets	MO = 11 NC = 15	↓ Kinetic factor V_max_ and *K*_m_, PRP 5-HT levels
(Pradalier et al., 1983) [217]	5-HT	Platelets	MO = 19 NC = 15	Unconjugated serotonin concentration was decreased during the attacks and decrease in 5-HT uptake by platelets, with a fall in Km and Vmax
(Kruglak et al., 1984) [218]	5-HT	Platelets	M = 18 NC = 26	Increased aggregability during attacks
(Lechner et al., 1985) [219]	5-HT, EN, ADP	Optical density method	M =14 NC = 30	↑ enhanced platelet aggregation and platelet sensitivity in migraine patients
(Lingjaerde, 1986) [220]	5-HT	Platelets in platelet rich-plasma (PRP). This method measures Km and Vmax	MO = 14 MA = 10 NC = 25	The size of the granular compartment was larger for classic than for common migraine, and the efflux rate from compartment III was
(Shukla et al., 1987) [221]	5-HT	Platelets	TTH = 20 M = 19 NC = 15	↑ uptake of 5-HT in platelets
(Kozubski et al., 1987) [222]	5-HT	Platelets	M = 12 NC = 10	↑ Increased number and affinity of fibrinogen receptors on the platelet surface in migraineurs.
(Joseph et al., 1988) [223]	5-HT	Plasma	MO = 7 MA = 5 NC = 6	Abnormal sensitivity to PAF
(D'Andrea et al., 1989a) [224]	5-HT, DA, E, NE,	Platelets obtained from PRP from whole blood HPLC colourimetric detection	MO = 19 MA = 9 NC = 15	High NE levels and low 5-HT/NE ratio in platelets of patients with MA platelet dense body hyposecretion
(D'Andrea et al., 1989b) [225]	5-HT	Platelets	MO = 19 MA = 9 NC = 15	Adhesion, surface activation, aggregation were investigated. Platelet dense bodies were increased. Increase in the content of serotonin, not evident in MA
(Herman et al., 1989) [226]	5-HT	Platelet aggregation was monitored using PRP, in a computerized four-channel aggregometer (CARAT)	M = 10 NC = 9	PAF may be involved in the 'pathogenesis of migraine and suggest that specific PAF receptor antagonists or synthesis blockers may help in the therapy of this disease
(Takeshima et al., 1989) [227]	5-HT	Cold pressor test. Blood was sampled three times through the three-way cock: before the stimulus, 1 min after the start of the stimulus, and 5 min after the start of the stimulus	MCH = 15 M = 13 NC = 14	Both PF4 and NE increased significantly, whereas FFA showed no remarkable changes. The increase of PF4, however, was independent of NE increase and FFA changes
(Riddle et al., 1989) [228]	5-HT	Electron microscope	MO = 21 MA = 15 NC = 15	Demonstrated structural alterations and increase in the number of dense bodies
(Kovacs et al., 1990) [179]	5-HT	Platelets in platelet rich-plasma (PRP)	MO = 17 MA = 14 NC = 13	Migraineurs have altered platelet aggregation properties and PAF may play a role. The EC50 value for ADP and platelet-activating factor were significantly higher.
(Govitrapong et al., 1992) [229]	5-HT	Platelets	M = 12 NC = 12	↓ in the maximal number of receptors (Bmax) on platelet membrane of migraine patients when compared to the normal healthy group
(Cotrim et al., 1993) [230]	5-HT	Platelet membrane fluidity was measured using the fluorescent probe TMA-DPH	MO = 11 MA = 20 NC = 17	Migraine have decreased membrane fluidity
(Jensen, 1994) [231]	5-HT	Platelet poor-plasma (PPP)	TTH = 13 NC = 29	Increased 5HT during attacks
(Kitano et al., 1994) [232]	5-HT	Platelets	MO = 19 MA = 10 TH = 22 NC = 27	↑ 11-dehydrothromboxane B2, platelet hyperfunction
(Jarman et al., 1996) [233]	5-HT	Platelets [^14^C]5HT release measured by Lingjaerde and Monstad method	M = 8 NC = 10	No change in release of platelet 5-HT
(Fioroni et al., 1996) [234]	5-HT, 5-HIAA, MAO-B	Platelets	M = 7 NC = 8	↓ reduced 5-HT and ↑ increased 5HIAA in luteal phase, suggesting a greater susceptibility
(Srikiatkhachorn, 1996) [235]	5-HT	Platelets in platelet rich-plasma (PRP)	M = 20 NC = 20	↓ Kinetic factor V_max_ and *K*_m_, = uptake of 5-HT
(Pukhal'skaya et al., 1998) [236]	5-HT	Platelets in platelet rich-plasma (PRP)	M = 8 NC = 8	5-HT transport systems in migraine patients and healthy donors are different
(Oishi, 1998) [237]	5-HT	Platelets	ETH = 10 CTH = 10 NC = 10	↑ platelet factor 4, D-thromboglobulin, thromboxane B, and 1 1-dehydrothromboxane B, concentrations in the plasma were significantly higher in the episodic tension- type headache group
(Srikiatkhachorn, 1998) [238]	5-HT	Platelets	M = 10 AAG = 10 NC = 10	Excessive use of analgesics depletes 5-HT from its storage sites and results in the hyposerotonergic state.
(Sarchielli et al., 2004) [239]	5-HT	Platelet-activating factor (PAF) HPLC radioimmunoassay method, internal jugular venous blood	MO = 5	↑ production of PAF levels no change in activity of PAF acetyl-hydrolase (PAF-AH), enzyme involved in the catabolism of this phospholipid mediator, in migraine attacks
(Yucel et al., 2014) [240]	5-HT	Blood fibrinogen, D-dimer, galectin-3 determined by ELISA	M = 59 NC = 30	↑ levels of fibrinogen, D-dimer, galectin-3 in migraine patients ↑ higher D-dimer levels during migraine attacks may indicate hypercoagulability

*AAG* Analgesic Abuse Group, *CDH* chronic daily headache, *CH* Cluster headache, *CM* complicated migraine, *CMF* chronic migraine with fibromyalgia, *MA* migraine with aura, *MO* migraine without aura, *NC* normal control, *PAF* platelet-activating factor, *PPP* platelet-poor plasma, *PRP* platelet-rich plasma, *TH* Tension headache; ↑ Increase; ↓ Decrease; = Unchanged

**Table 4:** Selected large scale biochemical studies of platelet serotonin in migraine patients and controls

Reference	Substance	Material and methods	Cases/ controls	Results
(Couch, 1976) [241]	5-HT	Platelet aggregation was tested to 1.7uM adenosine diphosphate employing light transmission methods modified after Born	M = 33 NC = 33	↑ platelet aggregability was measured by a) grading aggregation curves and b) by measuring percent disaggregation 3 min after peak aggregation was less
(Couch, 1977) [242]	5-HT	Platelets, optical density methods	M = 46 NC = 46	↑ migraine patients demonstrate platelet hyperaggregability, lower threshold for the platelet-release reaction and increased platelet stickiness following aggregation
(Deshmuck, 1977) [243]	5-HT	Platelets	M = 27 NC = 35	Platelet adhesiveness to glass beads and platelet aggregation response to ADP, epinephrine, thrombin and serotonin increased during the prodrome phase of migraine
(Manotti et al., 1983) [244]	5-HT	ADP induced platelet aggregation, ADP threshold concentration, platelet malondialdehyde production stimulated by thrombin, Beta-Thromboglobuli level in PPP	M = 30 NC = 30	Significant activation of platelet function
(Waldenlind et al., 1985) [245]	Platelets	Platelets	CH = 33 M = 34 NC = 50	↓ Kinetic factor V_max_ and *K*_m_, = uptake of 5-HT
(Buttinelli et al., 1985) [246]	5-HT	Platelet Aggregate Ratio (PAR) studied by Wu and Hoak’s technique	MO = 37 MA = 20 CM = 16 NC = 90	↑ circulating platelet aggregates
(Walkowiak et al., 1989) [247]	5-HT	Platelets and radioimmunoassay	MO = 34 NC = 28	Migraineurs have a higher receptor capacity for fibrinogen in platelets activated by ADP
(Joseph et al., 1989) [248]	5-HT	Platelets	M = 66 NC = 64	Increased number of dense bodies; altered coupling of 5-HT secretion from dense bodies and ionised calcium; decreased serotonin secretion.
(Ribeiro et al., 1990) [249]	5-HT, 5-HIAA	Serum serotonin (5-HT) measured by HPLC-EC	MO = 58 MA = 43 TH = 10 NC = 39	Significant decrease in Bmax, which suggests down-regulation of 5-HT2 receptors
(Jha et al., 1992) [250]	5-HT	Platelets	MA = 40	↑ platelet aggregation during the aura and headache phase of the migraine attack
(Leira et al., 1993) [251]	5-HT	Platelets in platelet rich-plasma (PRP)	TTH = 30 NC = 20	No change in PRP 5-HT levels
(D'Andrea et al., 1994) [178]	5-HT	Platelet-rich plasma (PRP) Serotonin was measured by HPLC and platelet factor 4 (PF4) with an enzyme immunoassay kit	MO = 41 MA = 62 NC = 26	Increased basal platelet 5-HT and increased 5-HTsecretion induced by both collagen and
(D'Andrea et al., 1995) [252]	5-HT	Platelet-rich plasma (PRP)	MO = 41 MA = 62 TH = 28 NC = 26	Plasma and platelet 5-HT peak in MM in ovulatory phase; 5-HT peak evident in follicular phase in TH and controls.
(Allais et al., 1997) [253]	5-HT	Platelet aggregation was stimulated by ADP 1 μM during the luteal phase of the menstrual cycle in Menstrual migraine patients	MM = 46 NC = 27	↑ platelet aggregation during luteal phase of the menstrual cycle
(Zeller et al., 2005) [254]	5-HT	Platelets during attack free interval	MO = 48 MA = 25 NC = 72	MA patients ↑ numbers of aggregates MO patients ↑ numbers of platelet-leucocyte aggregation and activation-dependent epitope expression
(Taffi et al., 2005) [255]	5-HT	Platelets in platelet rich-plasma (PRP). Membrane Na+/K + −ATPase activity and fluidity were determined with the fluorescent probes TMA-DPH and DPH	MO = 57 NC = 35	Migraine patients show intercritic changes in platelet membrane fluidity and activity that may be related to the oxidative stress caused by increased ONOO– levels
(Yucel et al., 2014) [240]	5-HT	Blood fibrinogen, D-dimer, galectin-3 determined by ELISA	M = 59 NC = 30	↑ levels of fibrinogen, D-dimer, galectin-3 in migraine patients ↑ higher D-dimer levels during migraine attacks may indicate hypercoagulability

*CH* cluster headache, *CM* complicated migraine, *MA* migraine with aura, *MM* menstrual migraine, *MO* migraine without aura, *PPP* platelet-poor plasma, *PRP* platelet-rich plasma, *TH* Tension headache, *NC* normal control; ↑ Increase; ↓ Decrease; = Unchanged

For the most part, investigations report increased platelet activation and it is argued that these changes could reflect parallel biochemical changes in the CNS (see Tables 3 and 4). Patterns of platelet behaviour/activation have also been found to differ in migraine subgroups, a finding that could indicate different pathomechanisms [178, 179]. There is, however, no platelet phenotype that can be used as a direct diagnostic biomarker for migraine [180]. It is difficult to draw final conclusions for platelet biology studies in migraine as they have investigated different aspects of platelet biology and have at times reported contrasting results. The general state of affairs is that platelet anomalies are not considered to be causal for migraine and that this topic merits additional investigation to gain a fuller understanding [181].

#### Biochemical studies of serotonin in plasma

Data regarding serotonin levels in plasma of migraineurs should be treated with caution due to differing definitions of plasma serotonin and determination of serotonin content in plasma. This is because normal blood plasma is composed of two distinguishable fractions namely platelet-rich-plasma (PRP) and platelet-poor plasma (PPP) [182]. Platelet-rich-plasma is blood plasma that is mostly bound to platelets whereas platelet-poor plasma refers to the fraction of freely circulating serotonin which is said to be distinct to PRP. This fraction of blood plasma contains a very low number of platelets and is identified as the extraplatelet pool [183]. This portion of plasma is difficult to assay consistently owing to the difficulty of preparing PPP without causing release of serotonin from platelets and without platelet contamination. Additionally, whether or not a study is using PPP or a less refined plasma subset is often unclear, adding to uncertainty [183]. This is perhaps demonstrated in the contrasting results that have been obtained with a few studies reporting elevated levels of plasma serotonin, specifically studies by Dvilansky et al., 1976, Ferrari et al., 1989 and Milovanovic et al., 1999 [184–186] (see Tables 3 and 4). Conversely studies by Curran et al., [187], Rydzewski, [188] and Nagata et al., [189] reported lower levels of plasma serotonin in migraineurs than in controls during the attack. More consistency and standardised methods of assaying blood plasma in biochemical studies are required in order to move forward.

#### Biochemical studies of serotonin in urine

In addition to the studies examining serotonin metabolism in CSF, saliva, platelets and plasma there have been several studies using urine as a proxy for serotonin activity in the brain. These were often the first studies and were fundamental in highlighting a possible serotonin-dependent mechanism urging further investigation [17]. Four out of seven independent studies observed an increase in the urinary excretion of the main metabolite of serotonin, 5-hydroxyindoleacetic acid (5-HIAA), in association with migraine attacks [17, 149, 190, 177] (see Table 5). Two studies [186, 191] reported a decrease in urinary excretion of 5-HIAA and one study reported no difference [192]. Bousser et al., [191] provided evidence of a 31% lowered urinary 5-HIAA excretion in 44 young adult female migraine patients between attacks and in 33 healthy age- and sex-matched control subjects [191]. Milovanovic et al., [173] reported a similar trend of lowered urinary 5-HIAA excretion although the sample size in this study was smaller consisting of a total of 18 migraine patients [186]. Despite urine’s ease of access, only a small number of studies have thus far investigated the biochemistry of urine in migraineurs.

**Table 5:** Biochemical studies of 5-hydroxyindoleacetic acid (5-HIAA) in the urine of migraine patients and controls

Reference	Migraine classification	Controls/cases	Sample tested	Levels	Controls/cases	Sample tested	Levels
(Sicuteri et al., 1961) [17]	Not reported	Controls: 15	Urine				
		M: 15		↑			
(Curran et al., 1965) [187]	Not reported	Controls: 10	Urine		Controls: 21	Plasma	
		M: 18		↑	M: 11		↓
(Curzon et al., 1966) [149]	Not reported	Controls: 4	Urine				
		M: 9		=			
(Kangasni et al., 1972) [149]	Not reported	Controls: 6	Urine		Controls: 6	CSF	
		M: 9		↑	M: 14		↑
(Deanovic et al., 1975) [190]	(Headache, 1962) [193]	Controls: 4	Urine				
		M: 14		↑			
(Bousser et al., 1986) [191]	(Headache, 1970) [194]	Controls: 33	Urine				
		M: 44		↓			
(Milovanovic et al., 1999) [186]	(ICHD-I, 1988) [195]	Controls: 11	Urine		Controls: 5	Plasma	
		M: 8		↓	M: 5		↑
		TTH: 10		↓	TTH: 7		

*M* migraine, *CSF* cerebrospinal fluid, *TTH* Tension-Type Headache; ↑ Increase; ↓ Decrease; = Unchanged

#### Biochemical model of serotonin

The existing data support the premise that serotonin is low interictally but increased ictally (during attacks themselves) in the migraine brain [143, 196]. According to this model, during an attack serotonin is released from platelets into blood plasma causing a short-lived increase in free plasma serotonin that raises serotonin levels in the brain [197]. The second arm of the model posits that raised levels of serotonin in the brain, during a migraine attack, exerts a vasoconstrictor effect on intracerebral arteries initiating the aura phase and cerebral ischemia with resulting visual disturbances. The concentration of serotonin eventually lowers because serotonin is broken down and excreted and this lowering post-attack is thought to produce vasodilation of extracerebral arteries causing headache and pain [197]. Migraine is generally regarded as a low serotonin syndrome. Diminished levels of serotonin in the brain lower the threshold for pain and are evident in some disorders co-morbid with migraine including chronic tension headache [198] and depression [199] as well as other chronic diseases such as fibromyalgia, irritable bowel syndrome [200], premenstrual dysphoric disorder and depression. In addition, tricyclic antidepressants can also reduce the frequency of migraine attacks by increasing serotonin signalling further supporting a role for serotonin in the pathobiology of migraine [201]. Serotonin has also been implicated in CSD and the trigeminovascular pathway. Experimental depletion of serotonin in rats, enhances the development of CSD with accompanying cerebrovascular changes and increases responsiveness and sensitivity of the trigeminal system [202–204].

Metabolomics is an emerging field that has recently shown that variations in brain metabolic homeostasis are associated with neurological disorders [205, 206]. In particular a recent study by Shyti et al., [191] identified metabolic changes by mass spectrometry in a transgenic mouse model of hemiplegic migraine after experimentally induced CSD [207]. This is the first study demonstrating CSD-induced metabolite differences in Familial Hemiplegic Migraine type 1 R192Q mice relative to WT mice and that these events can be captured beyond the CNS in plasma samples. Although we still do not know what the connection of central and peripheral serotonergic changes in migraine may be, further study based on metabolic profiling of biological fluids which contain the “products of numerous genome-wide and proteome-wide interactions” [208], the ‘metabolome’ [209] will continue to shed light on the inner workings of the migraine brain. To study serotonin deficiency-associated mechanisms a study by Weng et al., used a metabolomics approach to identify biomarkers from serotonin deficient mice [47]. The mice were depleted of serotonin via two approaches (1) use of p-chlorophenylalanine (pCPA) to pharmacologically inhibit tryptophan hydroxylase (Tph), a rate-limiting enzyme in serotonin biosynthesis and (2) genetic knockout of the Tph2 isoform. In this study serotonin deficiency was associated with altered energy metabolism and several biomarkers were identified in the serotonin deficient mice to be significantly altered compared with the control mice [47]. Moreover, the combination of sensitive brain imaging techniques and metabolomics platforms hold optimism to identify novel metabolite-gene relationships and new insight into small molecular changes in neuronal metabolism to enhance our understanding of biochemical pathways “functional readout”. Pharmacometabolomics-informed pharmacogenomics studies will therefore occupy an important position in the coming tide of molecular and “omics” research.

## Conclusion

The evidence so far points to migraine as a biochemically complex disorder involving several neurotransmitter systems which converge in their synaptic pathways. The general trend observed is that migraine patients have alterations of neurotransmitters in biological fluids with different biochemistry from controls, however the interpretation of the biological significance of these peripheral changes is unresolved. At present, despite the observation of biochemical alterations, specific diagnostic markers are lacking for this disorder. In addition MA and MO sufferers may exhibit varying degrees and types of neurotransmitter dysfunction due to differing neurological features of both migraine types which may be related to the aetiology and underlying genetic component of each migraine subtype. Insufficient serotonergic input due to changes in synthesis, release, receptor function/expression, reuptake and transport in the brain and throughout the body may contribute to vulnerability to migraine. Genetic studies have highlighted involvement of the serotonin transporter gene SERT in migraine susceptibility. However, serotonergic biochemistry is complex and evidently a multiplicity of factors, rather than any single one, combine to result in migraine phenotypes. More research is required to shed light on the course of these various biochemical phenomena and enable more robust conclusions on causal relationships. The absence of modern biochemical studies underscores the need for more rigorously conducted research utilizing larger sample sizes, updated IHS classification criteria and more sensitive bioassays and instruments and standardized methods for sample collection, preparation/analysis. Novel approaches for investigating the serotonergic system using PET imaging technology integrated with a metabolomics and systems biology platform may help to better understand the biochemical milieu and metabolome of migraineurs and establish if the biochemical changes are linked to the clinical presentation of the disease process. In conclusion, given the therapeutic efficacy of triptans, investigating the functionality of the serotonergic system in the migraine brain remains on the agenda to illuminate disease pathomechanisms and new lines of treatment to enable a superior understanding of the disease process that will further aid in the diagnosis, treatment and management of migraine and headache related disorders.

## Abbreviations

5-HIAA: 5-hydroxyindoleacetic acid
AAG: Analgesic abuse group
CDH: Chronic daily headache
CH: Cluster headache
CM: Complicated migraine
CMF: Chronic migraine with fibromyalgia
CSD: Cortical spreading depression
CSF: Cerebrospinal fluid
MA: Migraine with aura
MM: Menstrual migraine
MO: Migraine without aura
NC: Normal control
PPP: Platelet-poor plasma
PRP: Platelet-rich plasma
TH: Tension headache
